# Function of Oncogene *Mycn* in Adult Neurogenesis and Oligodendrogenesis

**DOI:** 10.1007/s12035-021-02584-7

**Published:** 2021-10-08

**Authors:** Jiao Chen, Zhonghui Guan

**Affiliations:** Department of Anesthesia and Perioperative Care, University of California San Francisco, San Francisco, CA 94143 USA

**Keywords:** *Mycn*, Adult, Neurogenesis, Oligodendrogenesis

## Abstract

Human *MYCN* is an oncogene amplified in neuroblastoma and many other tumors. Both human *MYCN* and mouse *Mycn* genes are important in embryonic brain development, but their functions in adult healthy nerve system are completely unknown. Here, with *Mycn*-eGFP mice and quantitative RT-PCR, we found that *Mycn* was expressed in specific brain regions of young adult mice, including subventricular zone (SVZ), subgranular zone (SGZ), olfactory bulb (OB), subcallosal zone (SCZ), and corpus callosum (CC). With immunohistochemistry (IHC), we found that many *Mycn*-expressing cells expressed neuroblast marker doublecortin (DCX) and proliferation marker Ki67. With *Dcx-creER* and *Mki67-creER* mouse lines, we fate mapped *Dcx*-expressing neuroblasts and *Mki67*-expressing proliferation cells, along with deleting *Mycn* from these cells in adult mice. We found that knocking out *Mycn* from adult neuroblasts or proliferating cells significantly reduced cells in proliferation in SVZ, SGZ, OB, SCZ, and CC. We also demonstrated that the *Mycn*-deficient neuroblasts in SGZ matured quicker than wild-type neuroblasts, and that *Mycn*-deficient proliferating cells were more likely to survive in SVZ, SGZ, OB, SCZ, and CC compared to wild type. Thus, our results demonstrate that, in addition to causing tumors in the nervous system, oncogene *Mycn* has a crucial function in neurogenesis and oligodendrogenesis in adult healthy brain.

**Supplementary Information:**

The online version contains supplementary material available at 10.1007/s12035-021-02584-7.

## Introduction

*MYCN*, the human gene encoding N-Myc, was first identified as an oncogene amplified in human neuroblastoma [1], a tumor characterized as having undifferentiated neuroblasts [2]. The expression level of *MYCN* correlates with the prognosis of neuroblastoma [3], and overexpression of human *MYCN* gene produces neuroblastoma in mice [4]. *MYCN* overexpression is also observed in other nervous system tumors such as medulloblastoma, glioblastoma, retinoblastoma, and spinal ependymoma, as well as tumors outside of the nervous system such as neuroendocrine prostate cancer, nephroblastoma (Wilms’ tumor), and many others [5–7]. The nucleotide sequence of *MYCN* is very similar to that of oncogene *MYC* [8].

In addition to causing tumors, *MYCN* is also expressed in human fetal brain [9], and heterozygous *MYCN* mutation causes Feingold syndrome characterized as reduced brain size and learning disability [10]. Similarly, mouse *Mycn* gene is expressed in embryonic and neonatal forebrains and hindbrains [11], and in embryonic eye, spinal cord, dorsal root ganglion (DRG) (spinal ganglia) and trigeminal ganglia (cranial ganglion) [12]. A homozygous *Mycn*-deficient mouse dies between 10.5 and 12.5 days of gestation, with significant developmental abnormalities in central and peripheral nervous systems [13]. Conditional deletion of *Mycn* from *Nestin*-expressing neuronal progenitor cells (NPCs) in embryo results in dramatic reduction of NPC proliferation and disruption of neuronal differentiation, leading to a substantial decrease in brain mass and disorganization of both the cortex and cerebellum [14]. *Mycn* also plays an important role in the proliferation and survival of embryonic sympathetic neuroblasts [15]. Finally, *Mycn* is highly functional in pluripotent cells as well, because overexpression of *Mycn*, along with *Oct4*, *Sox2*, and *Klf4*, transforms mouse embryonic fibroblasts into induced pluripotent stem cells [16].

As *Mycn* was initially found to be not expressed in adult brain [11], the function of *Mycn* in the nervous system of an healthy adult has never been revealed. With *Mycn*-EGFP reporter mice [17], we found that *Mycn* is expressed in restricted regions in adult brain, such as subventricular zone (SVZ), subgranular zone (SGZ) of hippocampal dentate gyrus (DG), the two major sites for adult neurogenesis [18], and corpus callosum (CC) and subcallosal zone (SCZ), a site of adult oligodendrogenesis [19]. Our results further demonstrate that *Mycn* plays important roles in adult neurogenesis and oligodendrogenesis.

## Materials and Method

### Animals

All animal procedures were performed according to protocols approved by Institutional Animal Care and Use of Laboratory animals. Animals were housed in groups of five in a pathogen-free barrier facility. Young adult (8–12-week-old) male and female mice were used in all experiments. Tamoxifen or hydroxytamoxifen was injected in doses of 100 mg per kg intraperitoneally.

*Mycn*-EGFP mouse, STOCK Tg(Mycn-EGFP)ET250Gsat/Mmucd, RRID:MMRRC_010553-UCD, was obtained from the Mutant Mouse Resource and Research Center (MMRRC) at University of California at Davis, an NIH-funded strain repository, and was donated to the MMRRC by Nathaniel Heintz, Ph.D. at Rockefeller University to GENSAT.

*Mycn* fl/fl mice were obtained from Jackson Laboratory (B6,129-Mycn ^tm1Psk^/J, stock No. 006933); *Mki67*-creER (Mki67 ^tm2.1(cre/ERT2)cle^/J, stock No. 029803). *Dcx*-creER was a gift from professor Zhiqi Xiong. *Rosa26*-TdTomato-loxp-STOP-loxp mice were obtained from Jackson Laboratory (B6. Cg-Gt (ROSA)26Sor ^tm14(CAG−Td−Tomato)Hze^/J, stock No. 007914).

### Immunohistochemistry

Animals were sacrificed and then intracardially perfused with ice-cold PBS and 4% paraformaldehyde (PFA; Thermo Scientific, AC416785000). Brains were dissected, post-fixed in 4% paraformaldehyde overnight, and embedded in OCT compound (Tissue-Tek, Thermo Fisher, 4585). Coronal brain sections (20 µm thick) were collected by cryostat. The sections were washed with 1 × PBS and incubated with blocking buffer with 10% fetal bovine serum (FBS) (Thermo Fisher) and 0.3% Triton X-100 in PBS for 30 min at room temperature. Primary antibodies diluted in 1% FBS and 0.3% Triton X-100 in PBS were added to the slices and incubated overnight at 4 °C. Subsequently, conjugated secondary antibodies of Alexa Fluor 488, Alexa Fluor 555, or Alexa Fluor 647 (1:1000; Invitrogen) were added, and DAPI (1:1000; Invitrogen) was used for showing nucleuses.

The following primary antibodies and their dilutions were used: GFAP (rabbit, 1:1000; Dako, #Z0334), Ki67 (rabbit, 1:1000; Abcam), doublecortin (DCX) (rabbit, 1:500; Abcam), NeuN (mouse, 1:500; Millipore), oligodendrocyte transcription factor 2 (Olig2) (rabbit, 1:500; Millipore, #AB9610), and Tbr2 (rabbit, 1:500; Abcam). Images were captured using a Zeiss LSM700 confocal microscope as a single plain and were processed with Fiji/ImageJ (NIH).

### Cell Counting

For EdU + cell number counting, we chose three regions to count the EdU + cell number, including SVZ, hippocampus, and olfactory bulb. For each region, we cut the brain from the same region and collected 20-µm cryosections of each brain region from three animals per group. In order to get the information in the whole region, we mounted, immunostained, and counted the EdU + cells in every sixth section of the brain region. To quantify the Td-tomato + cells in the targeted regions, we counted Td-tomato + cells from 3 to 4 animals per group in slices with the same region. We use the number of cells per field to measure the density of cells within SVZ, CC, SGZ, SCZ, and olfactory bulb (OB), with the field defined as the specific brain regions under 20 × magnification, and count the Td-Tomato + cell number in that specific region. To analyze the distribution of the newborn cells in dentate gyrus, we divided the granule cell layer into three layers equally, as shown in Fig. 7b, counted the Td-tomato–positive cells in each layer, and calculated the percentage of the cell number in each layer versus the whole Td-tomato + cell number in the dentate gyrus. To quantify the Td-tomato–positive cells that co-express Olig2, we counted Td-tomato–positive cells in three sections in each area per animal and quantified the double-labeled Td-tomato/Olig2–immunoreactive cells.

### EdU Staining

EdU solution (10 mg/kg; Invitrogen, C10337) was injected intraperitoneally 24 h before tissue collection. Click-iT reaction cocktail (1 × Click-iT reaction buffer, CuSO_4_, Alexa Fluor azide, and 1 × reaction buffer additive) was prepared and applied to slides for 30 min and washed with PBS for 10 min three times, followed by first antibody incubation overnight. Then, the slides were incubated with second antibody for 1 h at room temperature and washed with PBS for 10 min, followed by DAPI staining.

### Flow Cytometry

The brain region such as SVZ was freshly microdissected and homogenized. The homogenized tissue was filtered then mixed with Percol (the final concentration of Percol is 30%). After being centrifuged at 800 g for 20 min, the cells were harvested for the subsequent flow cytometry. Cells were first incubated with anti-mouse CD16/32 in PBS for 5 min to block the Fc receptor. Cells were then washed with PBS and incubated with near-IR (1:1000; BioLegend) for 30 min. Cells were washed with PBS and resuspended in the desired antibody mix in PBS and stained for 30 min at 4 °C. After washing with PBS, cells were fixed and permeabilized using the Foxp3 transcription factor staining buffer kit (Thermo Fisher) for intracellular and intranuclear staining and washed two times with Perm/Wash (0.01% sodium azide, 0.5% saponin, and 2% BSA in PBS). Cells were then resuspended in PBS and analyzed. Flow cytometry was performed using a FACS Symphony A5 (BD Biosciences) and analyzed with the FlowJo software. Cell sorting was performed using a FACSAria III. The following fluorochrome-conjugated monoclonal antibodies were used: Ki67 (clone SOLA15, 1:5000) and DCX (anti-rabbit, 1:100; Abcam). Dead cells and duplets were excluded for analysis using SSC-A/H, FSC-A/H, and a fixable viability kit (near-IR staining; BioLegend).

### qRT-PCR

For qRT-PCR, cDNA synthesis was performed using a SuperScript III First-Strand Synthesis System (Invitrogen, 18,080–051). Quantitative PCR was performed using PerfeCTa SYBR Green FastMix Rox (Quanta Biosciences) in a qPCR system (Bio-Rad). β-Actin was used as the housekeeping gene for normalization. The following primers were used: *Actb* (β-actin), CCACACCCGCCACCAGTTCG/TACAGCCCGGGGAGCATCGT; *Mycn*, AGCGTTCAACTAGCAGACCAT/CGAAAGGGCAGATTGTGTGG; and *Dcx*, CGACCAAGACGCAAATGGAAC/CAATGACAGCGGCAGGTACA.

### Experimental Design and Statistical Analysis

The experiments were conducted with 3–4 animals each group, the minimal number of animals to obtain results with statistical significance. For immunohistochemistry (IHC) studies, at least 3 sections from each animal were immuno-stained, and the average value of all the sections from the same animals was used to represent the value of that particular animal. For flow cytometry studies, one experiment was performed in one animal. For qRT-PCR, three duplicates were performed for each primer pair, and the average value of all three duplicates was used to represent the value of that particular animal. No data was excluded, and data are expressed as mean ± SEM. Statistical analysis was performed using GraphPad Prism, version 8.0. Unpaired two-tailed Student’s *t* tests were used for single comparisons between two groups. Other data were analyzed using one-way or two-way analysis of variance (ANOVA).

## Results

### *Mycn* Is Expressed in Adult Healthy Brain of Mice

**Fig. 1:**
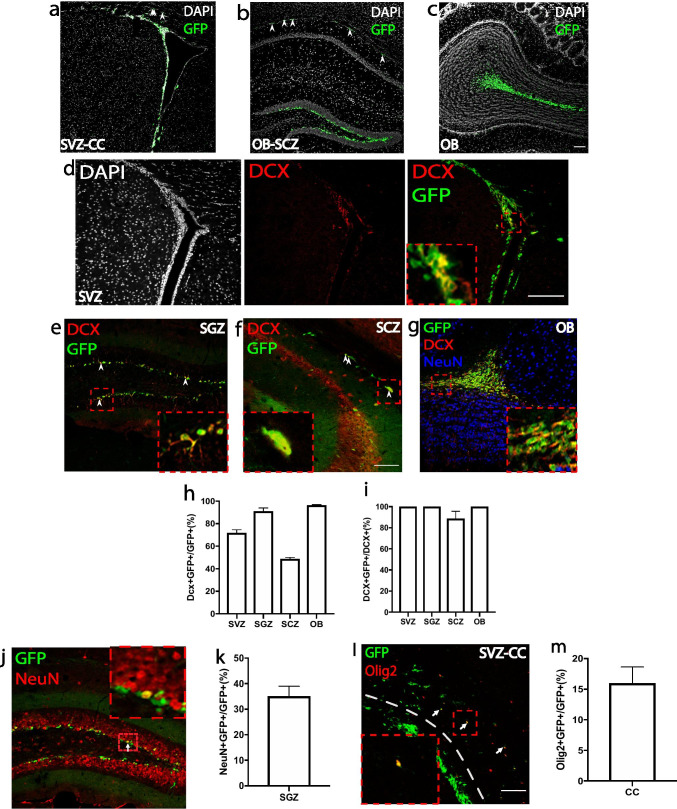
*Mycn* is expressed in specific sites in adult mouse brain. **a**–**c** IHC of GFP and DAPI in the subventricular zone (SVZ) and corpus callosum (CC) (**a**), subgranular zone (SGZ) and subcallosal zone (SCZ) (**b**), and olfactory bulb (OB) (**c**) of young adult *Mycn-eGFP* reporter mice. The arrowheads point to the GFP-positive cells in CC (**a**) and SCZ (**b**). **d** IHC of DAPI, DCX, and GFP in the SVZ of *Mycn-eGFP* reporter mice. The enlarged image shows the co-localization of GFP and DCX in SVZ. **e**–**g** IHC of DCX and GFP in the SGZ (**e**), SCZ (**f**), and olfactory bulb (**g**) of *Mycn-eGFP* reporter mice. The arrowheads point to the DCX and GFP double–positive cells (**e**, **f**), and the enlarged images show the GFP and DCX double–positive cells. **h** Quantification of **d**–**g** showing the percentage of DCX/GFP double–positive cells among GFP ( +) cells in the SVZ, SGZ, SCZ, and OB of young adult *Mycn-eGFP* reporter mice. **i** Quantification of **d**–**g** showing the percentage of DCX/GFP double–positive cells among DCX ( +) cells in the SVZ, SGZ, SCZ, and OB of young adult *Mycn-eGFP* reporter mice. **j** IHC of GFP and NeuN in the dentate gyrus of young adult *Mycn-eGFP* reporter mice. The arrow points to the GFP and NeuN double–positive cells, and the enlarged figure shows the NeuN and GFP double–positive cells. **k** Quantification of **j** showing the percentage of NeuN/GFP double–positive cells among GFP ( +) cells in the SGZ of *Mycn-eGFP* reporter mice. **l** IHC of GFP and Olig2 in the CC above SVZ of *Mycn-eGFP* reporter mice. The dashed line shows the boundary between SVZ and CC, and the arrows point to the GFP and Olig2 double–positive cells, with the enlarged figure showing the Olig2 and GFP double–positive cell. **m** Quantification of **i** showing the percentage of Olig2/GFP double–positive cells among GFP ( +) cells in the CC of young adult *Mycn-eGFP* reporter mice

To investigate if *Mycn* is expressed in adult brain, we studied young adult (8–12 weeks old) *Mycn*-eGFP reporter mice generated by GENSAT [17], in which eGFP is expressed under the control of bacterial artificial chromosome containing the promoter and the enhancers of mouse *Mycn* gene. We found that GFP was expressed in SVZ (Fig. 1a), SGZ (Fig. 1b), and olfactory bulb (OB) (Fig. 1c). We also found GFP ( +) cells in CC (Fig. 1a, arrowheads), as well as in SCZ (Fig. 1b, arrowheads), a thin region between the hippocampus and CC that contains neural stem cells [19]. We then dissected the SVZ and hippocampus from *Mycn*-eGFP reporter mice and sorted out the GFP ( +) cells from the SVZ, dentate gyrus, and SCZ with fluorescence-activated cell sorting (FACS). With qRT-PCR, we confirmed that *Mycn* gene was enriched in the GFP ( +) cells in SVZ (Supplementary Fig. 1a), dentate gyrus (Supplementary Fig. 1b), and SCZ (Supplementary Fig. 1c).

**Fig. 2:**
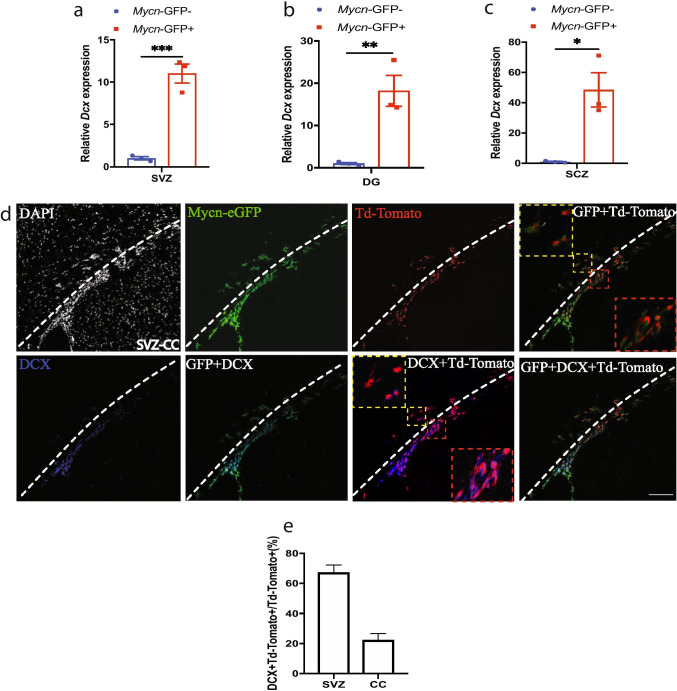
*Mycn* is enriched in *Dcx*-expressing cells. **a**–**c** In *Mycn-eGFP* reporter mice, qRT-PCR study shows that the GFP ( +) cells sorted by FACS from SVZ, dentate gyrus (DG), and SCZ had higher *Dcx* mRNA levels than GFP ( −) cells. Data are shown as mean ± SEM, and the analysis performed was unpaired two-tailed Student’s *t* test. **d** IHC of GFP, Td-Tomato, and DCX in SVZ and CC from a *Dcx-creER/* + *; Td-Tomato/* + *; Mycn-eGFP* mouse 1 day after hydroxytamoxifen treatment. In these mice, the cells expressing *Dcx* in the last 24 h were labeled with Td-Tomato. The dashed lines show the boundary between SVZ and CC. Virtually, all the Td-Tomato ( +) cells in SVZ and CC were GFP ( +). Most Td-Tomato ( +) cells in SVZ and some Td-Tomato ( +) cells in CC were also DCX ( +). Enlarged figures show the GFP/Td-Tomato double–positive cells and the GFP/DCX double–positive cells. Scale bar = 100 µm. **e** Quantification of **d** showing the percentage of DCX/Td-Tomato double–positive cells among Td-Tomato ( +) cells in the SVZ and CC of a *Dcx-creER/* + *; Td-Tomato/* + *; Mycn-eGFP* mouse 1 day after hydroxytamoxifen treatment. Data are shown as mean ± SEM, and the analysis performed was unpaired two-tailed Student’s *t* test

Because *Mycn* is associated with neuroblastoma, we were particularly interested in finding out whether *Mycn* is expressed in the neuroblasts of adult healthy mice. Our IHC studies of brain sections of young adult *Mycn*-eGFP reporter mice with DCX, which is expressed in adult neuroblast and neuronal intermediate progenitor cells (nIPCs) [20], revealed the co-localization of DCX with GFP in SVZ (Fig. 1d), SGZ (Fig. 1e), SCZ (Fig. 1f), and OB (Fig. 1g). In fact, 72% of GFP ( +) cells in SVZ, 91% of GFP ( +) cells in SGZ, 49% of GFP ( +) cells in SCZ, and 96% of GFP ( +) cells in OB co-expressed DCX (Fig. 1h), whereas almost all DCX ( +) cells in these regions were GFP ( +) (Fig. 1i). We also found that 35% of the GFP ( +) cells in the dentate gyrus of young adult *Mycn*-eGFP reporter mice also expressed NeuN (Fig. 1j, k). On the other hand, 16% of GFP ( +) cells in CC co-expressed Olig2, a marker of oligodendrocyte lineage cells [21] (Fig. 1l, m). We did qRT-PCR from sorted GFP ( +) cells of young adult *Mycn*-eGFP reporter mice and found that GFP ( +) cells had significantly higher expression of *Dcx* gene than GFP ( −) cells in SVZ (Fig. 2a), dentate gyrus (Fig. 2b), and SCZ (Fig. 2c).

To further investigate *Mycn* expression in adult neuroblasts, we crossed a *Mycn-eGFP* mouse with a *Dcx-creER* mouse [22] and with a Td-Tomato reporter mouse [23]. In the young adult *Dcx-creER/* + *; TdTomato/* + *; Mycn-eGFP/* + mice 1 day after hydroxytamoxifen treatment, the cells that expressed *Dcx* in the past 1 day were fate labeled by TdTomato (Fig. 2d). In these mice, we found both GFP ( +) and TdTomato ( +) cells in SVZ and CC, and virtually all TdTomato ( +) cells were GFP ( +) (Fig. 2d), suggesting that *Mycn* was expressed in cells with either current *Dcx* expression or recent history of *Dcx* expression in this region. Further IHC with DCX antibody showed that 67% of TdTomato ( +) cells in SVZ and 22% of TdTomato ( +) cells in CC were DCX ( +) and thus continued to express DCX (Fig. 2d, e).

Our IHC studies with young adult *Mycn*-eGFP reporter mice also showed that 6% of the GFP ( +) cells in SVZ, 17% of GFP ( +) cells in SGZ, and 56% of GFP ( +) cells in SCZ also expressed another neuroblast marker (Tbr2), encoded by gene *Eomes* [20] (Supplementary Fig. 1d–g). Moreover, our analysis of the published single-cell RNA-Seq (scRNA-Seq) dataset from perinatal, juvenile, and adult dentate gyrus [20] shows that *Mycn* is expressed in radial glia-like (RGL) cells, nIPCs, neuroblasts, and immature granule cells (GCs), as well as in some mature GCs (Supplementary Fig. 2a), consistent with our IHC study that some GFP ( +) cells in the dentate gyrus of *Mycn*-eGFP mice expressed the mature neuron marker NeuN (Fig. 1j, k). Interestingly, the expression pattern of *Mycn* is very similar to that of *Dcx* in dentate gyrus, which is also expressed in RGLs, nIPCs, neuroblasts, immature GCs, and some mature GCs (Supplementary Fig. 2b). In contrast, the neuroblast marker *Eomes* is expressed more specifically in nIPCs and neuroblasts (Supplementary Fig. 2c). On the other hand, our analysis of published scRNA-Seq dataset from oligodendrocyte lineage cells of juvenile and adult CNS [24] shows that *Mycn* is expressed in some oligodendrocyte precursor cells (OPCs), differentiation-committed oligodendrocyte precursors (COPs), and certain myelin-forming oligodendrocytes (MFOLs) and mature oligodendrocytes (MOLs) (Supplementary Fig. 3a). In comparison, *Olig2* gene is expressed in every oligodendrocyte lineage cells (Supplementary Fig. 3b).

### *Mycn*-Expressing Cells Can Proliferate

**Fig. 3:**
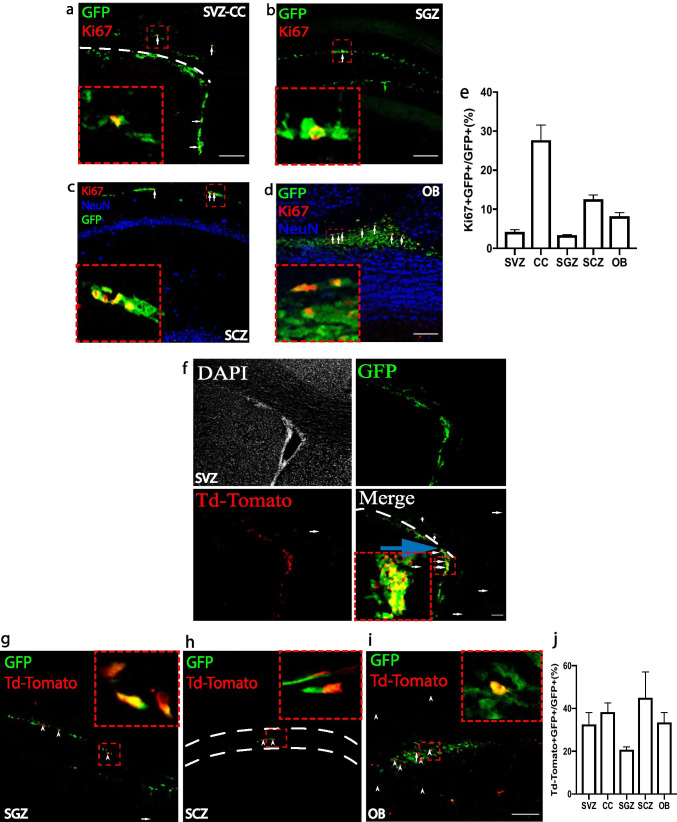
*Mycn*-expressing cells can proliferate. **a**–**d** IHC of GFP and Ki67 in the SVZ and CC (**a**), SGZ (**b**), SCZ (**c**), and OB (**d**) of a young adult *Mycn-eGFP* mouse. The dashed line in **a** shows the boundary between SVZ and CC. The arrows point to the GFP and Ki67 double–positive cells, with the enlarged figures showing the Ki67 and GFP double–positive cells. **e** The quantitative analysis of **a**–**d** shows the percentage of Ki67 ( +) cells in GFP ( +) cells in different brain regions of a *Mycn*-eGFP reporter mouse. **f**–**i** IHC of GFP and Td-Tomato in a *Mki67-creER/* + *; Td-Tomato/* + *; Mycn-eGFP* mouse in SVZ and CC (**f**), SGZ (**g**), SCZ (**h**), and OB (**i**) after tamoxifen treatment for 5 days. In these mice, the proliferating cells and the cells proliferated in the past 5 days were labeled with Td-Tomato. The dashed line in **f** shows the boundary between SVZ and CC, and the dashed lines in **h** show the boundary of SCZ. The arrows and arrowheads show the GFP and Td-Tomato double–positive cells, and the enlarged figures show the Td-Tomato and GFP double–positive cells. **j** The quantitative analysis of **f**–**i** shows the percentage of GFP and Td-Tomato double–positive cells among GFP ( +) cells in the SVZ, CC, SGZ, SCZ, and OB of a *Mki67-creER/* + *; Td-Tomato/* + *; Mycn-eGFP* mouse after 5-day tamoxifen treatment. Scale bar = 100 µm

As one major character of adult neurogenesis and oligodendrogenesis is the proliferation of RGLs, nIPCs, neuroblasts, and OPCs [20, 21], we investigated if proliferation occurred in *Mycn*-expressing cells. Our IHC studies of brain sections from young adult *Mycn*-eGFP reporter mice with the proliferation marker Ki67 [25] showed that 4% of the GFP ( +) cells in SVZ, 28% of the GFP ( +) cells in CC, 3% of the GFP ( +) cells in SGZ, 13% of the GFP ( +) cells in SCZ, and 8% of the GFP ( +) cells in OB were Ki67 ( +) (Fig. 3a–e). To further confirm that *Mycn*-expressing cells do proliferate, we crossed the *Mycn*-eGFP mouse with the *Mki67-creER* mouse along with the Td-Tomato reporter mouse. In young adult *Mki67-creER/* + *; TdTomato/* + *; Mycn-eGFP/* + mice, tamoxifen treatment for 5 days fate labeled the proliferating cells and the cells recently proliferated within last 5 days with Td-Tomato. We found that in this animal, 33% of GFP ( +) cells in SVZ, 38% of GFP ( +) cells in CC, 21% of GFP ( +) cells in SGZ, 45% of GFP ( +) cells in SCZ, and 34% of GFP ( +) cells in OB were TdTomato ( +) (Fig. 3f–j). All together, these results suggest that *Mycn*-expressing cells in adult healthy brain proliferate in multiple brain regions.

### *Mycn* Is Essential for Cell Proliferation in Adult Neurogenesis and Oligodendrogenesis

**Fig. 4:**
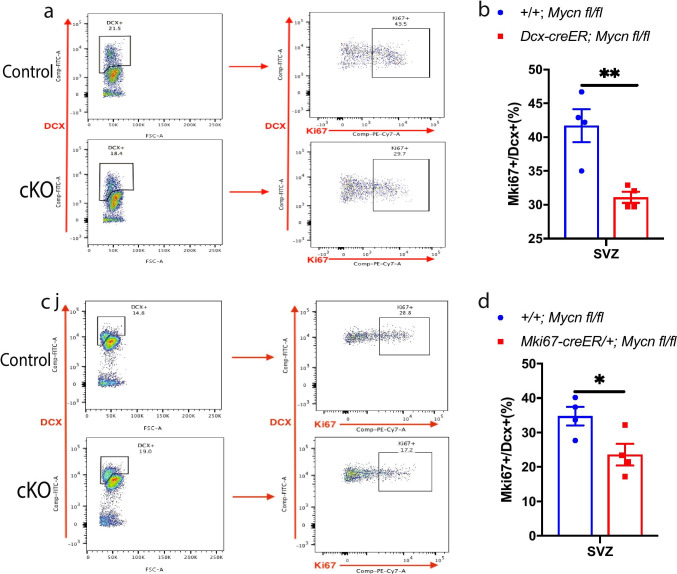
*Mycn* is required for the proliferation of DCX ( +) cells in SVZ. **a** Representative flow cytometry scatter plots to assess the proliferation of DCX ( +) cells in the SVZ of young adult *Dcx-creER* + */* + *; Mycn fl/fl* (control) versus *Dcx-creER/* + *; Mycn fl/fl* (cKO) mouse. **b** Quantification of **a** shows that the deletion of *Mycn* from *Dcx*-expressing cells significantly reduced the proliferation of DCX ( +) cells. **c** Representative flow cytometry scatter plots to assess the proliferation of DCX ( +) cells in the SVZ of young adult *Mki67-creER* + */* + *; mycn fl/fl* (control) versus *Mki67-creER/* + *; Mycn fl/fl* (cKO) mouse. **d** Quantification of **c** showing that the deletion of *Mycn* from *Mki67*-expressing cells significantly reduced the proliferation of DCX ( +) cells. Data are shown as mean ± SEM, and the analysis performed was unpaired two-tailed Student’s *t* test

To investigate if *Mycn* contributes to cell proliferation in adult neurogenesis, we crossed *Dcx*-creER line with *Mycn* flox line [14] to generate *Dcx-creER/* + *; Mycn fl/fl* mice. We treated young adult *Dcx-creER/* + *; Mycn fl/fl* and control + */* + *; Mycn fl/fl* mice with daily tamoxifen for 5 days to delete *Mycn* from any cells that have expressed *Dcx* in the past 5 days in *Dcx-creER/* + *; Mycn fl/fl* mice. With flow cytometry to analyze DCX ( +) cells in SVZ (Supplementary Fig. 4a, b), we found that the DCX ( +) cells with *Mycn* deletion had significant less proliferating Ki67 ( +) cells compared to DCX ( +) cells from control + */* + *; Mycn fl/fl* littermates (Fig. 4a, b). We also crossed *Mki67-creER* line with *Mycn* flox line to generate *Mki67-creER/* + *; Mycn fl/fl* mice, and daily tamoxifen treatment of young adult *Mki67-creER/* + *; Mycn fl/fl* and control + */* + *; Mycn fl/fl* mice for 5 days will delete *Mycn* from any cells that have expressed *Mki67* in the past 5 days in *Mki67-creER/* + *; Mycn fl/fl* mice. With flow cytometry (Supplementary Fig. 4c, d), we found that in SVZ, the deletion of *Mycn* from *Mki67*-expressing cells also resulted in significantly less proliferation of the DCX ( +) cells compared to that in control + */* + *; Mycn fl/fl* littermates (Fig. 4c, d). Of note, the proliferation rate of the total cell populations in SVZ also decreased in *Mycn* cKO animals compared with that in intact animals (Supplementary Fig. 5). These results suggest that *Mycn* contributes to the proliferation of DCX ( +) cells in SVZ in adult.

**Fig. 5:**
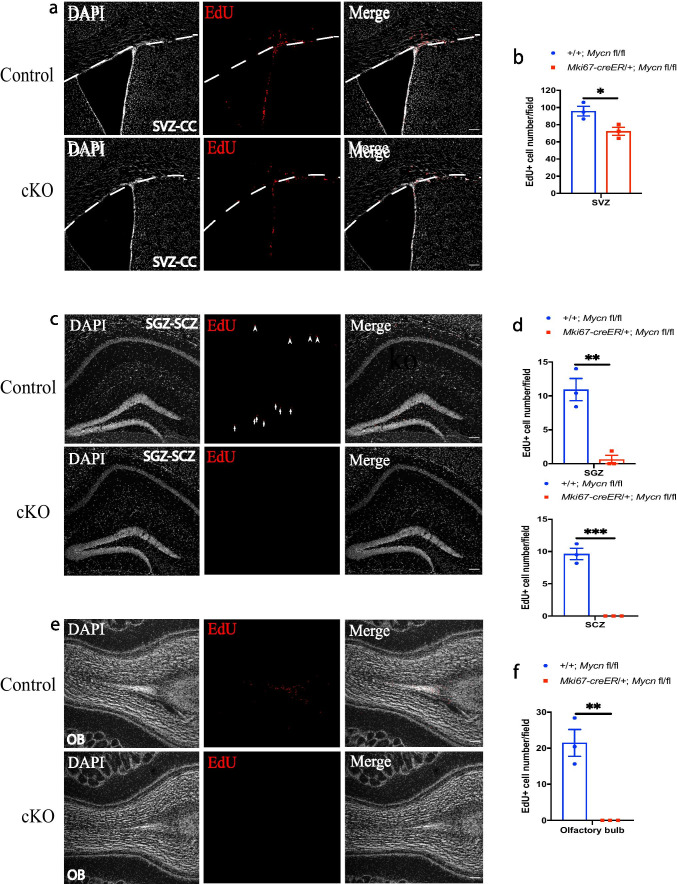
*Mycn* is required for cell proliferation in SVZ, SGZ, SCZ, and OB. IHC of EdU and DAPI in the SVZ (**a**), SGZ and SCZ (**c**), and OB (**e**) in young adult *Mki67-creER* + */* + *; Mycn fl/fl* (control) versus *Mki67-creER/* + *; Mycn fl/fl* (cKO) mouse. The dashed line shows the boundary between SVZ and CC (**a**). In **c**, the arrows point to the EdU-positive cells in SGZ, and the arrowheads point to the EdU-positive cells in SCZ. Scale bar = 100 µm. Quantification of **a**, **c**, and **e** shows that the deletion of *Mycn* from *Mki67*-expressing cells significantly reduced cell proliferation in SVZ (**b**), SGZ and SCZ (**d**), and OB (**f**). Data are shown as mean ± SEM, and the analysis performed was unpaired two-tailed Student’s *t* test

To further study the functional role of *Mycn* in cell proliferation in brain regions of young healthy animals, we injected EdU, a nucleoside analog of thymidine that can be incorporated into DNA during DNA synthesis for cell proliferation [26], into young adult *Mki67-creER/* + *; Mycn fl/fl* and control + */* + *; Mycn fl/fl* mice after daily tamoxifen treatment for 5 days. Consistent with our flow cytometry result (Fig. 4c, d), our IHC with EdU showed that deleting *Mycn* from *Mki67*-expressing cells significantly reduced the total number of EdU ( +)-proliferating cells in SVZ compared to that in control + */* + *; Mycn fl/fl* mice (Fig. 5a, b). Remarkably, although many EdU ( +) cells were observed in SGZ, SCZ, and OB in control + */* + *; Mycn fl/fl* mice, almost no EdU ( +) cells were observed in these regions when *Mycn* was deleted from *Mki67*-expressing cells (Fig. 5c–f). These results suggest that *Mycn* is required for cell proliferation in the sites of adult neurogenesis (SVZ and SGZ) and oligodendrogenesis (SCZ).

### *Mycn* Inhibits the Maturation of *Dcx*-Expressing Cells in Adult Dentate Gyrus

**Fig. 6:**
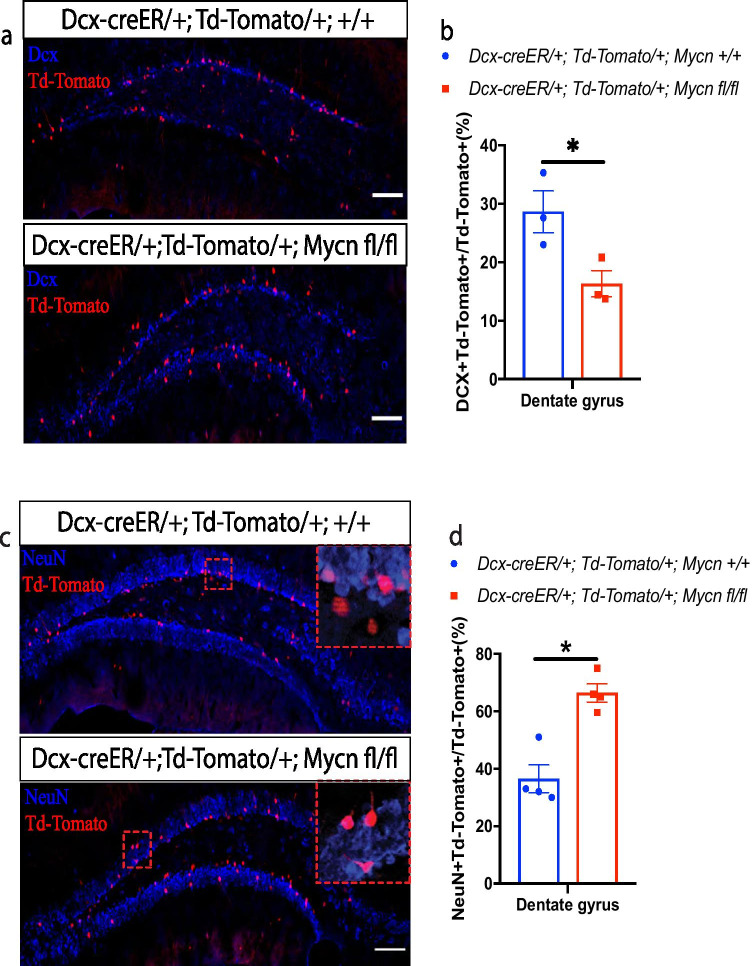
*Mycn* inhibits the maturation of *Dcx*-expressing cells in dentate gyrus. **a** IHC of DCX and Td-Tomato in the dentate gyrus of young adult *Dcx-creER/* + *; Td-Tomato/* + *;* + */* + (control) versus *Dcx-creER/* + *; Td-Tomato/* + *; mycn fl/fl* (cKO) mice on day 14 after the 5-day tamoxifen treatment. In these mice, the cells that had expressed *Dcx* 14–19 days ago were fate mapped with Td-Tomato. **b** Quantification of **a** shows that the *Dcx-creER/* + *; Td-Tomato/* + *, mycn fl/fl* (cKO) mice had significantly less DCX ( +) cells among the Td-Tomato ( +) cells compared to the control *Dcx-creER/* + *; Td-Tomato/* + *;* + */* + mice. **c** IHC of NeuN and Td-Tomato in the dentate gyrus of young adult *Dcx-creER/* + *; Td-Tomato/* + *;* + */* + (control) versus *Dcx-creER/* + *; Td-Tomato/* + *; mycn fl/fl* (cKO) mice on day 14 after the 5-day tamoxifen treatment. The enlarged figures show the NeuN and Td-Tomato double–positive cells. **d** Quantification of **c** shows that the *Dcx-creER/* + *; Td-Tomato/* + *; mycn fl/fl* (cKO) mice had significantly more NeuN ( +) cells among the Td-Tomato ( +) cells compared to the control *Dcx-creER/* + *; Td-Tomato/* + *;* + */* + mice. Scale bar = 100 µm. Data are shown as mean ± SEM, and the analysis performed was unpaired two-tailed Student’s *t* test

To investigate if *Mycn* is involved in maturation of neuroblast in adult, we crossed *Dcx*-creER line with TdTomato reporter line and *Mycn* flox line. Treating young adult and *Dcx-creER/* + *; TdTomato/* + *; Mycn fl/fl* mice and control *Dcx-creER/* + *; TdTomato/* + *;* + */* + mice with daily tamoxifen for 5 days fate labeled all the cells that have expressed *Dcx* in last 5 days with TdTomato, along with deletion of *Mycn* in TdTomato-labeled cells in *Dcx-creER/* + *; TdTomato/* + *; Mycn fl/fl* mice. We did IHC of dentate gyrus with DCX antibody 14 days after tamoxifen treatment and measured the percentage of TdTomato ( +) cells that are still DCX ( +). We found significantly less DCX ( +) cells in the TdTomato ( +) cells with *Mycn* deletion compared to the control TdTomato ( +) cells with intact *Mycn* (Fig. 6a, b), suggesting that TdTomato ( +) cells with *Mycn* deletion were more mature than TdTomato ( +) cells with intact *Mycn*. In addition, in the same mice, we observed that more TdTomato ( +) cells in the dentate gyrus of *Mycn*-deleted TdTomato ( +) cells expressed the mature neuron marker NeuN compared to that of control TdTomato ( +) cells with intact *Mycn* (Fig. 6c, d), further supporting that *Mycn* inhibits the maturation of *Dcx*-expressing cells.

**Fig. 7:**
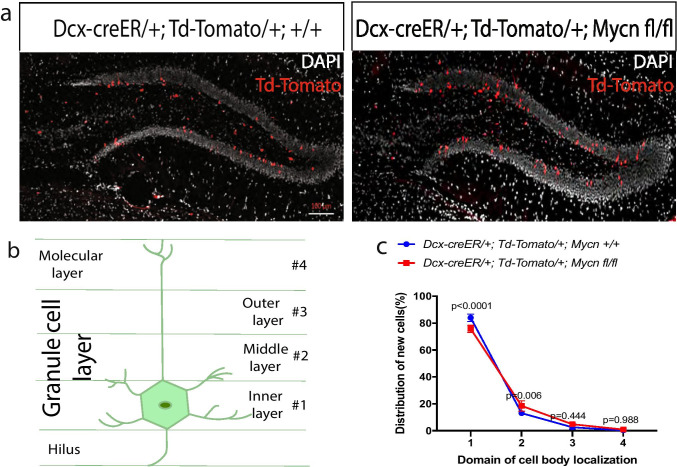
*Mycn* inhibits the migration of *Dcx*-expressed cells in dentate gyrus. **a** The distribution of Td-Tomato + cells in the dentate gyrus of young adult *Dcx-creER/* + *; Td-Tomato/* + *;* + */* + (control) versus *Dcx-creER/* + *; Td-Tomato/* + *; mycn fl/fl* (cKO) mice on day 14 after the 5-day tamoxifen treatment. In these mice, the cells that had expressed *Dcx* 14–19 days ago were fate mapped with Td-Tomato. **b** A model for dividing the dental gyrus granular layer into 4 areas. **c** Quantitative analysis of **a** shows the distribution of Td-Tomato ( +) cells in different dentate gyrus granular layers. Significantly more Td-Tomato ( +) cells had migrated from domain 1 into domain 2 in the *Dcx-creER/* + *; Td-Tomato/* + *; mycn fl/fl* (cKO) mice compared to the control *Dcx-creER/* + *; Td-Tomato/* + *;* + */* + mice. Data are shown as mean ± SEM, and the analysis performed was two-way ANOVA

Using the same type of animals, we also measured the distribution of TdTomato ( +) cells in the granule layer of dentate gyrus, because the newly generated neurons get more and more mature when they migrate from inner layers to outer layers [20, 27]. We found that 14 days after tamoxifen treatment of young adult and *Dcx-creER/* + *; TdTomato/* + *; Mycn fl/fl* mice and control *Dcx-creER/* + *; TdTomato/* + *;* + */* + mice, more TdTomato ( +) cells with *Mycn* deletion migrated away from the inner layer to the middle layer of dentate gyrus compared to the control TdTomato ( +) cells with intact *Mycn* (Fig. 7). Taken together, these results indicate that *Mycn* inhibits the maturation of neuroblasts in adult dentate gyrus.

### *Mycn* Inhibits the Survival of Newly Generated Cells in Sites of Adult Neurogenesis and Oligodendrogenesis

To monitor the fate of proliferated cells in the sites of adult neurogenesis and oligodendrogenesis, we crossed *Mki67-creER* line with TdTomato reporter line and *Mycn* flox line. Treating young adult *Mki67-creER/* + *; TdTomato/* + *; Mycn fl/fl* mice and control *Mki67-creER/* + *; TdTomato/* + *;* + */* + mice with daily tamoxifen for 5 days fate labeled all the cells that have expressed *Mki67* in last 5 days with TdTomato, along with deletion of *Mycn* from TdTomato-labeled cells in *Mki67-creER/* + *; TdTomato/* + *; Mycn fl/fl* mice. Fourteen days after the last tamoxifen treatment in the control *Mki67-creER/* + *; TdTomato/* + *;* + */* + mice, 94% of TdTomato ( +) cells in SVZ were DCX ( +) but none of them were NeuN ( +) (Supplementary Figs. 6a and 7), whereas in dentate gyrus, 65% of TdTomato ( +) cells were DCX ( +) and 72% of TdTomato ( +) cells were NeuN ( +) (Supplementary Figs. 6b and 7a, b), and in OB, 36% of TdTomato ( +) cells were DCX ( +) and 63% of TdTomato ( +) cells were NeuN ( +) (Supplementary Figs. 6c and 7a, b). These results suggest that most of the proliferating cells in SVZ, dentate gyrus, and OB develop into neuroblasts or mature neurons. On the other hand, 66% of TdTomato ( +) cells in SCZ and 83% of TdTomato ( +) cells in CC were Olig2 ( +) (Supplementary Figs. 6d, e and 7c), suggesting that the cells which proliferated 2 weeks ago mainly developed into oligodendrocyte lineage cells in SCZ and CC. Very little, if any, of the TdTomato ( +) cells in these regions were microglia or astrocytes (Supplementary Fig. 6).

**Fig. 8:**
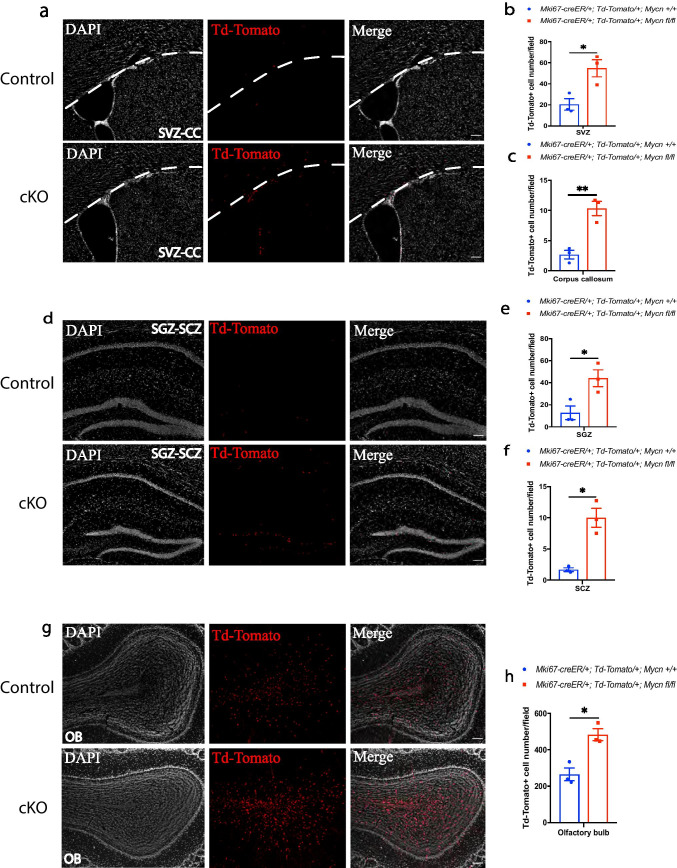
*Mycn* inhibits the survival of newly generated cells in young adult brain. Fate mapping of newly generated Td-Tomato ( +) cells in the SVZ and CC (**a**), dentate gyrus and SCZ (**d**), and OB (**g**) of young adult *Mki67-creER/* + *; Td-Tomato/* + *;* + */* + (control) versus *Mki67-creER/* + *; Td-Tomato/* + *; Mycn fl/fl* (cKO) mouse 14 days after the 5-day tamoxifen treatment. In these mice, the cells that had expressed *Mki67* 14–19 days ago were fate mapped with Td-Tomato. The dashed lines in **a** show the boundary between SVZ and CC. Scale bar = 100 µm. Quantification of **a**, **d**, and **g** shows that the deletion of *Mycn* from *Mki67*-expressing cells significantly enhanced the number of newly generated cells in SVZ (**b**), CC (**c**), dentate gyrus (**e**), SCZ (**f**), and OB (**h**). Data are shown as mean ± SEM, and the analysis performed was unpaired two-tailed Student’s *t* test

We then compared the TdTomato ( +) cells between *Mki67-creER/* + *; TdTomato/* + *; Mycn fl/fl* mice and control *Mki67-creER/* + *; TdTomato/* + *;* + */* + mice 14 days after the last tamoxifen treatment and found that animals with *Mycn* deletion in *Mki67*-expressing cells had significantly more TdTomato ( +) cells than the control animals in SVZ and CC (Fig. 8a–c), SGZ and SCZ (Fig. 8d–f), and OB (Fig. 8g, h), suggesting that *Mycn* inhibits the survival of newly generated cells in these regions.

## Discussion

Human *MYCN* is an oncogene involved in many tumors, including neuroblastoma, which is characterized as inappropriate proliferation of undifferentiated neuronal progenitor cells [5]. The copy numbers of *MYCN* are closely associated with the aggressiveness of neuroblastoma [28], with increased expression of *MYCN* that increases the proliferative potential of neuroblastoma cells [29]. Inhibition of MYCN function suppresses the proliferation and enhances the neuronal differentiation of *MYCN*-amplified neuroblastoma cells [30], and with different conditions, *MYCN* can either promote or inhibit apoptosis of neuroblastoma cells [5]. In addition to tumorigenesis, *MYCN* is also crucial for embryonic development, as it is expressed in human fetal brain [9], and heterozygous *MYCN* mutation causes microcephaly and learning disability in human [10]. Similarly, mouse *Mycn* is also expressed in embryonic central and peripheral nervous systems, and homozygous *Mycn* mutant mouse dies between 10.5 and 12.5 days of gestation [12, 13]. Deletion of *Mycn* from embryonic neuronal stem cells severely reduces the proliferation of embryonic neuronal progenitor cells and dramatically increases neuronal differentiation, with limited effect on apoptosis of embryonic neuronal progenitor cells [14]. On the other hand, overexpression of *Mycn* in mouse multipotent sympathoadrenal progenitors promotes proliferation, apoptosis, and neural lineage commitment [31]. In this study, we demonstrated that *Mycn* plays a crucial role for adult neurogenesis and oligodendrogenesis in mice.

### *Mycn* Is Expressed in Adult Brain

With *Mycn*-EGFP reporter mice, we found that *Mycn* is expressed in SVZ (Fig. 1a) and SGZ (Fig. 1b), two major sites of adult neurogenesis, as well as in OB (Fig. 1c), CC (Fig. 1a), and SCZ (Fig. 1b). Our further analyses revealed that many *Mycn*-EGFP cells co-expressed the neuroblast marker DCX in SVZ, SGZ, SCZ, and OB (Fig. 1d–g), as well as the proliferation marker Ki67 in SVZ, SGZ, OB, SCZ, and CC (Fig. 3a–e). Some *Mycn*-EGFP cells were also found in the granular layer of dentate gyrus, and these cells were also NeuN ( +) (Fig. 1j), suggesting that *Mycn* was expressed in certain hippocampal neurons, like those newly differentiated from neuroblasts. In addition, some *Mycn*-EGFP cells in CC also co-expressed the oligodendrocyte marker Olig2 (Fig. 1l, m), indicating that *Mycn* was expressed in oligodendrocyte lineage cells. Indeed, analysis of the published scRNA-Seq result from adult mouse dentate gyrus [20] showed that *Mycn* is mainly expressed in nIPCs, neuroblasts, and immature GCs and in small portions of the mature GDs (Supplementary Fig. 2a), similar to the expression pattern of *Dcx* (Supplementary Fig. 2b). Interestingly, although almost all DCX ( +) cells in these regions co-expressed GFP in *Mycn*-EGFP reporter mice (Fig. 1j), not all GFP ( +) cells were DCX ( +) (Fig. 1i). As the DCX ( +) cells were detected by DCX IHC in this study, some cells with a relatively low level of *Dcx* expression might not be detected by DCX IHC.

### *Mycn* Is Required for the Proliferation of Adult nIPCs and Neuroblasts

By deletion of *Mycn* from adult *Dcx*-expressing cells or in adult *Mki67*-expressing proliferating cells, we demonstrated that *Mycn* is required for the proliferation of nIPCs and neuroblasts in SVZ, SGZ, and OB. Unlike in SVZ where the proliferation is mildly reduced in *Mycn* cKO animals (Fig. 5b), in SGZ and OB, the proliferation is almost completely prevented in *Mycn* cKO animals (Fig. 5d, f), suggesting that *Mycn* is absolutely required for the proliferation of nIPCs and neuroblasts in SGZ and OB, whereas the proliferation in SVZ has *Mycn*-independent mechanism. The effect of *Mycn* on the proliferation of adult nIPCs and neuroblasts is likely mediated by the axis of cyclin-dependent kinase (CDK)-retinoblastoma protein (RB)-E2F transcription factor family, a core transcriptional machinery that determines if a cell exits the G1 phase to enter the S phase of cell cycle [32], because CDK6, RB, and E2F1 are required for the proliferation of adult neuronal progenitors and immature newborn dentate granule cell neurons [33–35], and knockdown of *MYCN* resulted in downregulation of CDK6 and E2F1 in neuroblastoma cells [36]. Deletion of *Mycn* from embryonic neuronal progenitor cells or knockdown of *MYCN* from neuroblastoma cells also upregulates CDK inhibitor p27 [14, 36], which is known to modulate CDK6 kinase activity in adult hippocampus to control the expansion of neuronal progenitor cells [34].

### *Mycn* Inhibits the Maturation of Adult Neuroblasts and the Migration of Newly Generated Neurons

With the fate mapping of *Dcx*-expressing cells in dentate gyrus, we found that the *Dcx*-expressing cells with *Mycn* deletion matured faster than the control *Dcx*-expressing cells (Fig. 6), and the newly generated granule neurons with *Mycn* deletion migrated further than the control neurons (Fig. 7), suggesting that *Mycn* inhibits the maturation of adult neuroblasts and the newly generated neurons. Similarly, knockdown *MYCN* promotes neuronal differentiation in neuroblastoma [37], and retinoic acid, which induces morphological differentiation of neuroblastoma cells [38], inhibits the expression of *MYCN* in these tumor cells [39].

### *Mycn* Inhibits the Survival of Adult Neuronal Progenitor Cells

With the fate mapping of *Mki67*-expressing proliferated cells in SVZ and CC (Fig. 8a–c), SGZ and SCZ (Fig. 8d–f), and OB (Fig. 8g, h) 14 days after they had expressed *Mki67*, we found that *Mycn* deletion from *Mki67*-expressing cells increased the number of the proliferated cells. The phenomenon of increased number of the proliferated cells was not the result of more cells being proliferated, because *Mki67*-expressing cells with *Mycn* deletion proliferated significantly less (Fig. 5). These results suggest that *Mycn* inhibits the survival of adult neuronal progenitor cells. The mechanism on how *Mycn* regulates the survival of adult neuronal progenitor cells needs to be further studied.

### *Mycn* Is Required for the Genesis of Adult Oligodendrocytes

In *Mycn*-EGFP reporter mice, we found GFP ( +) cells in CC (Fig. 1a) and SCZ (Fig. 1b), a known adult oligodendrogenesis site between hippocampus and CC [19]. Our fate-mapping study of *Dcx*-expressing cells revealed that these GFP ( +) cells in CC originate from the *Dcx*-expressing cells in SVZ (Fig. 2d), and our IHC study showed that some of the GFP ( +) cells in SCZ co-expressed DCX (Fig. 1f). Some of these GFP ( +) cells in CC and SCZ also co-expressed Ki67 (Fig. 3a, c), suggesting that they were proliferating cells, and our fate-mapping studies showed that the majority of *Mki67*-expressing cells in these regions developed into Olig2 ( +) oligodendrocytes (Supplementary Figs. 6d, e and 7c). In fact, some of the GFP ( +) cells in CC were Olig2 ( +) (Fig. 1l). These results suggest that the *Mycn*-expressing cells in these regions developed into oligodendrocyte lineage cells. Indeed, analysis of published scRNA-Seq from cells of oligodendrocyte lineage [24] showed that *Mycn* is expressed in OPCs, CDPs, and certain MFOLs and MOLs (Supplementary Fig. 3a).

We found that deletion of *Mycn* from *Mki67*-expressing cells blocked cell proliferation in SCZ (Fig. 5c, d), suggesting that *Mycn* is required for the cell proliferation in this region. In addition, with fate mapping of proliferated cells, we found significantly more survived cells that have recently proliferated in CC and SCZ of *Mycn* cKO animals (Fig. 8c, f). Thus, like in adult neurogenesis, *Mycn* promotes cell proliferation and inhibits cell survival in adult oligodendrogenesis sites. Interestingly, CDK4, rather than CDK6, is essential for the proliferation of adult oligodendrocyte progenitor cells [40], and knockdown of CDK4 in neuroblastoma cells reduces their proliferation [41]. In addition, both E2F1 [42] and p27 [43] contribute to the regulation of oligodendrocyte progenitor proliferation. Thus, it would be interesting to investigate the possible interaction between *Mycn* and CBK-BR-E2F pathway in adult oligodendrogenesis.

## Supplementary Information

Below is the link to the electronic supplementary material.

Supplementary file1 (DOCX 6948 kb)

## Data Availability

No data or material needs to be submitted.

## Code Availability

No code needs to be submitted.
