# Supplementary material for: Function of Oncogene Mycn in Adult Neurogenesis and Oligodendrogenesis
Source: Mol Neurobiol. 2021 Oct 8;59(1):77–92. doi: 10.1007/s12035-021-02584-7 (PMC8786763; doi:10.1007/s12035-021-02584-7)
Supplement: Supplementary file 1 — Supplementary file1 (DOCX 6948 kb) [file 12035_2021_2584_MOESM1_ESM.docx]

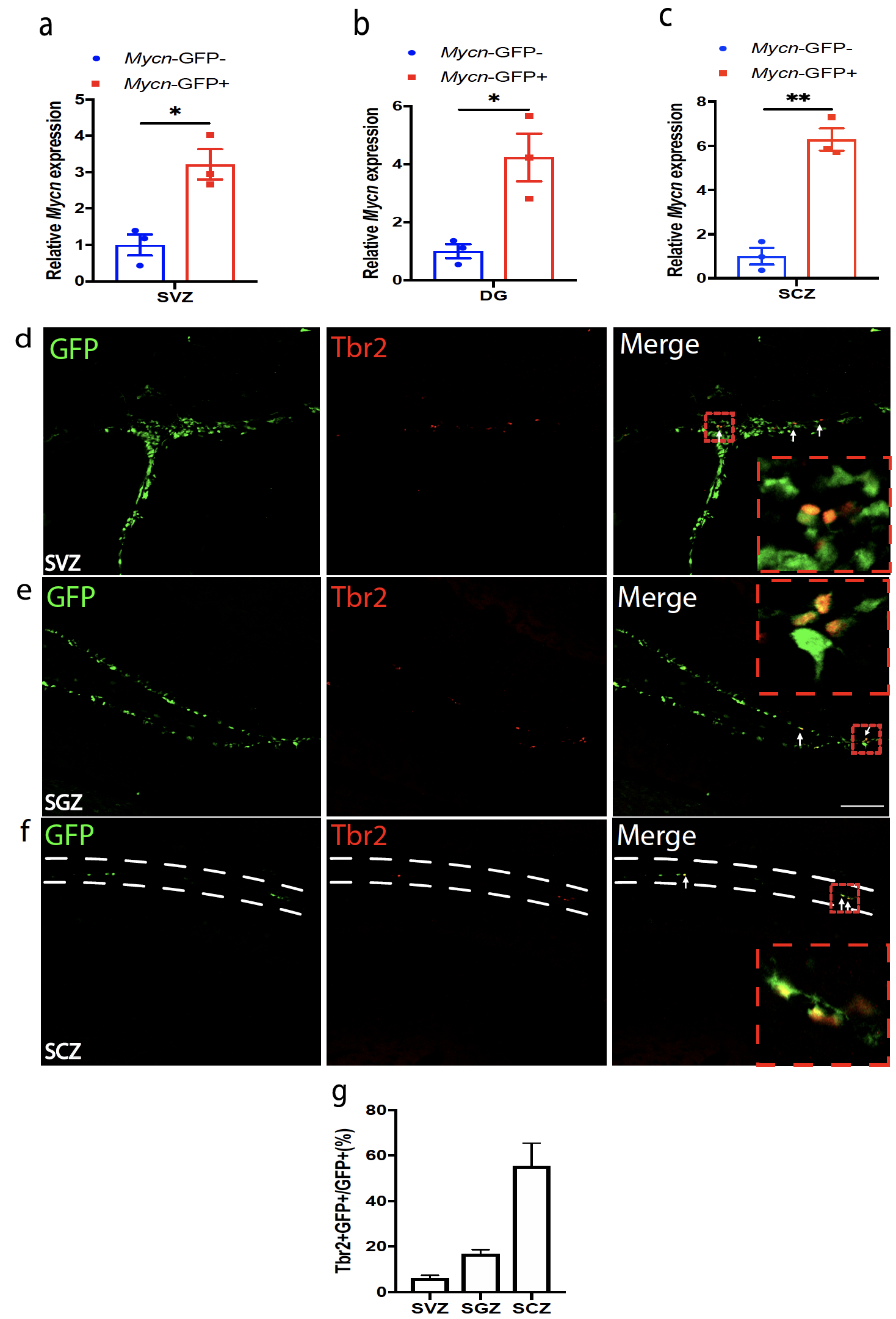


**Supplementary Figure 1. *Mycn* expression in adult mouse brain.**

(**a-c**) Compared to the GFP (-) cells, the *Mycn*-eGFP (+) cells sorted by FACS from SVZ (**a**), dentate gyrus (DG) (**b**) and SCZ (**c**) of young adult *Mycn-eGFP* reporter mice had higher level of *Mycn* mRNA. Data are shown as Mean ± SEM, and the analysis was unpaired two-tailed Student t-test.

(**d-f**) IHC of GFP and Tbr2 in SVZ (**d**), SGZ (**e**) and SCZ (**f**) of young adult *Mycn-eGFP* reporter mice. The dashed lines show the area of SCZ (i). The arrows point to the GFP and Tbr2 double positive cells, and the enlarged figures show the Tbr2 and GFP double positive cells. Scale bar = 100 μm.

(**g**) Quantification of (**d-f)** showing the percentage of Tbr2/GFP double positive cells among GFP (+) cells in SVZ, SGZ, SCZ of *Mycn-eGFP* reporter mice. Data are shown as Mean ± SEM.


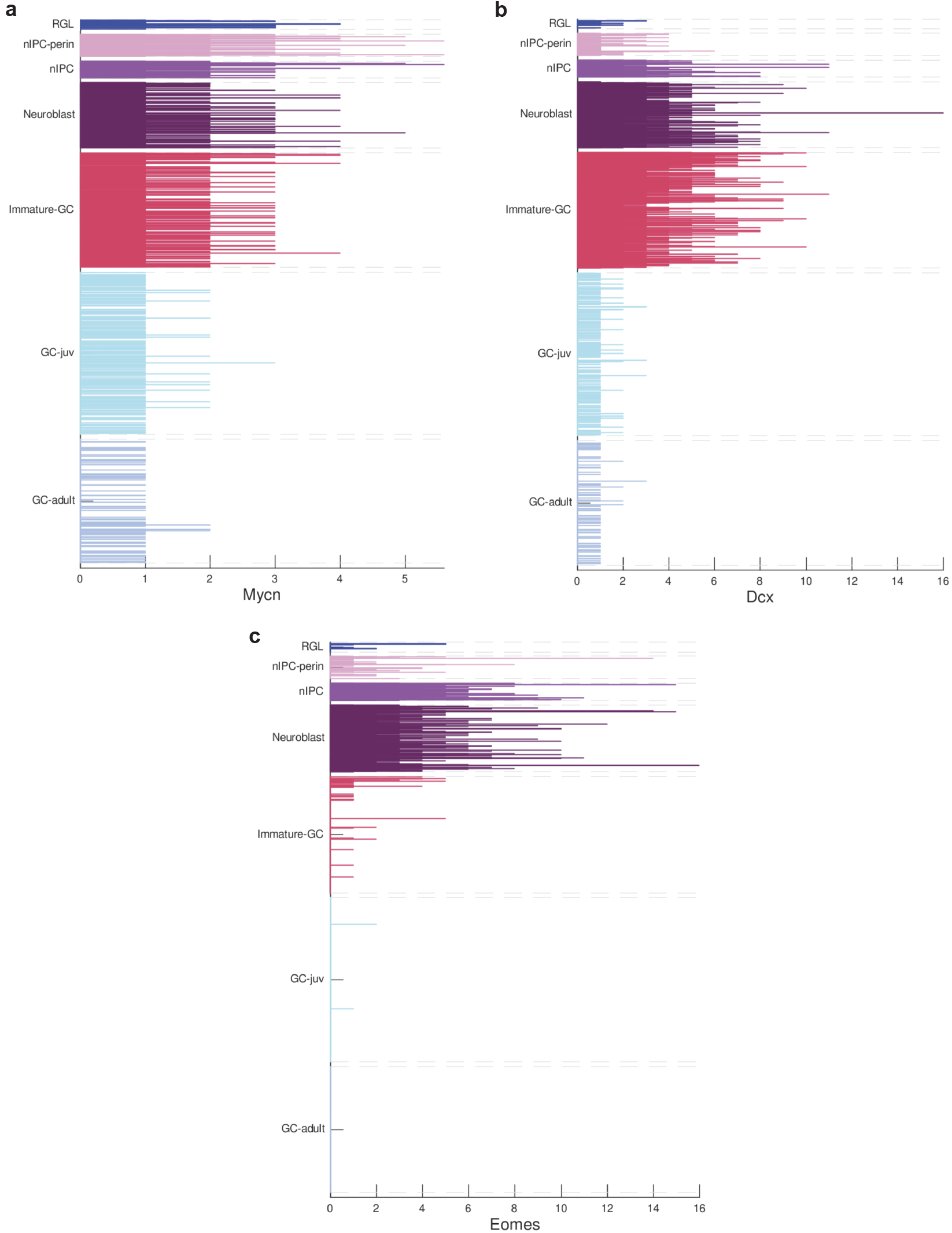
**Supplementary Figure 2. Expression of *Mycn*, *Dcx* and *Eomes* (Tbr2) in dentate gyrus.**

Single cell RNA sequencing results show that both *Mycn* (**a**) and *Dcx* (**b**) are expressed in radial glia-like cells (RGL), adult and perinatal neuronal intermediate progenitor cells (nIPC and nIPC-perin), neuroblast, immature granule cells (Immature-GC), juvenile granule cells (GC-juv), and some of the adult granule cells (GC-adult). In contrast, *Eomes* (**c**) is only expressed in RGL, nIPC-perin, nIPC and neuroblast.


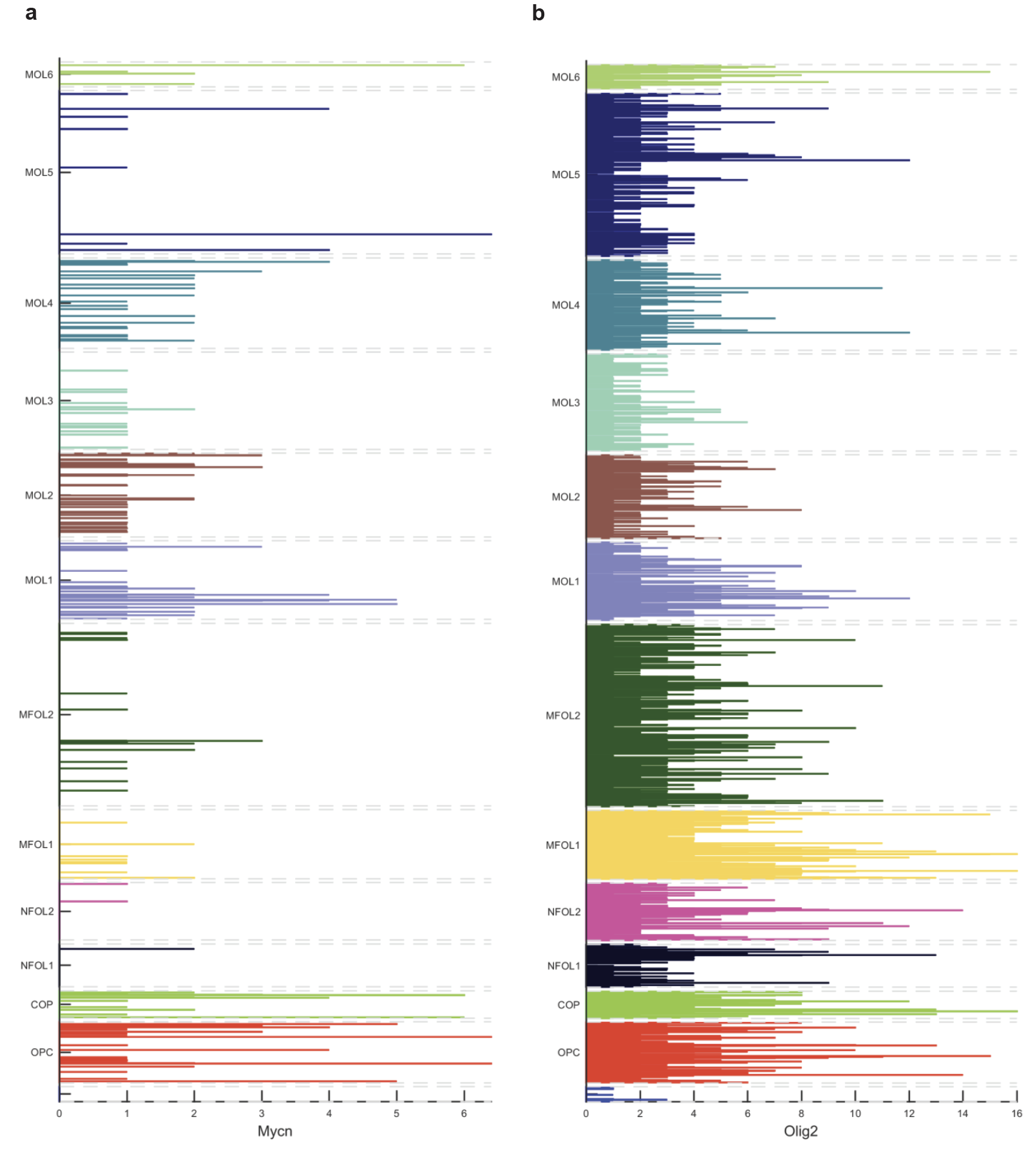


**Supplementary Figure 3. Expression of *Mycn* and *Olig2* in oligodendrocyte cell lineage.**

Single cell RNA Sequencing results show that *Mycn* (**a**) is expressed in some of the oligodendrocyte precursor cells (OPC), the differentiation-committed oligodendrocyte precursors (COP), myelin-forming oligodendrocytes (MFOL) and mature oligodendrocytes (MOL). In contrast, *Olig2* (**b**) is expressed in all oligodendrocyte lineage cells.

**Supplementary Figure 4.** Flow cytometry gating strategy of Fig. 3.

Flow cytometry scatter plots show population distribution in *+/+; Mycn fl/fl* (**a**) and *Dcx-creER/+; Mycn fl/fl* (**b**) in Fig. 3**a**, and *+/+; Mycn fl/fl* (**c**) and *Mki67-creER/+; Mycn fl/fl* (**d**) in Fig. 3**c**, and how the gates were drawn to select the DCX (+) cell population.
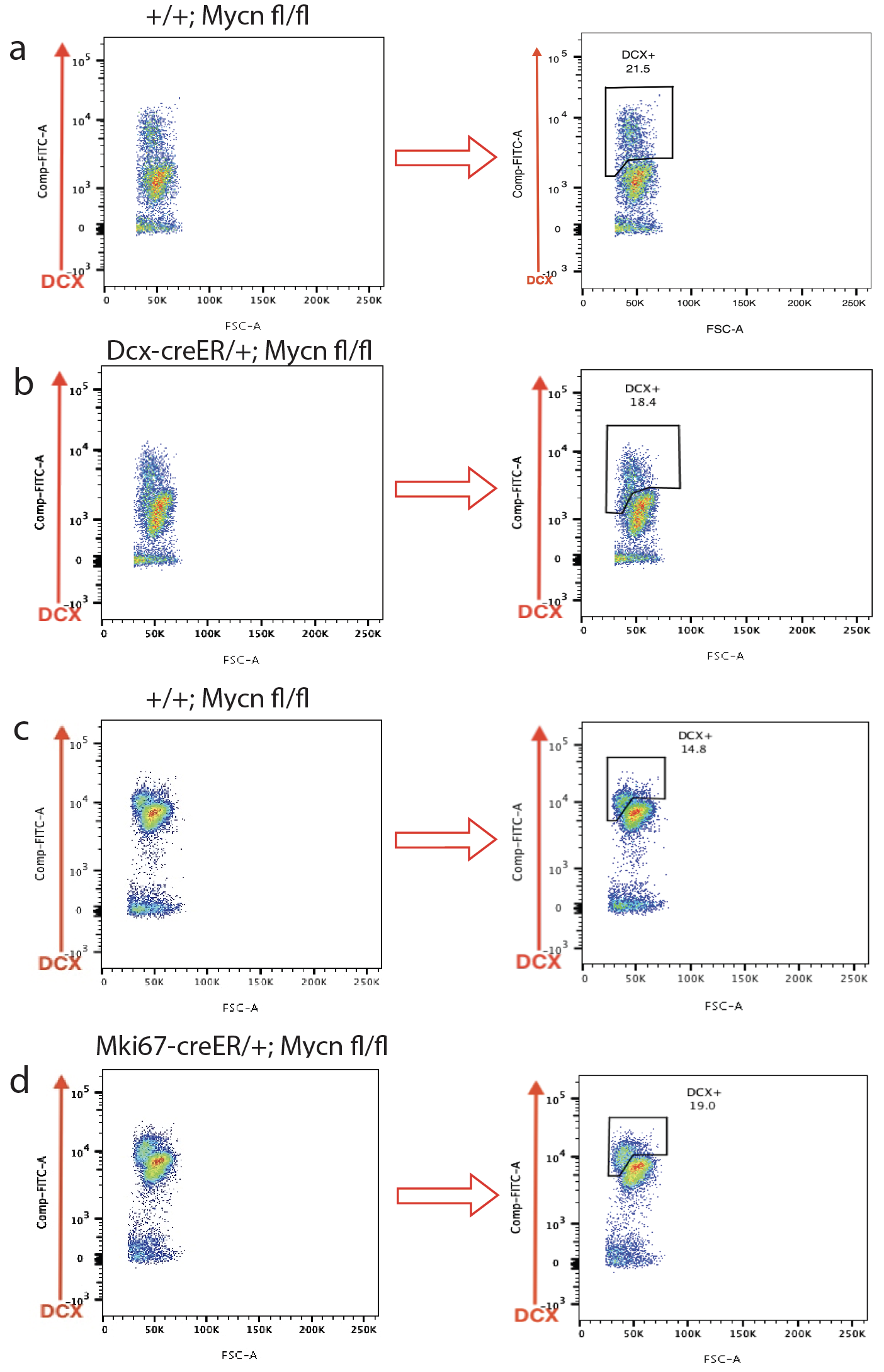
 The higher background signal in (**c**) and (**d**) was likely caused by the potential higher concentration of DCX antibody in the new batch of the same antibody as (**a**) and (**b**).


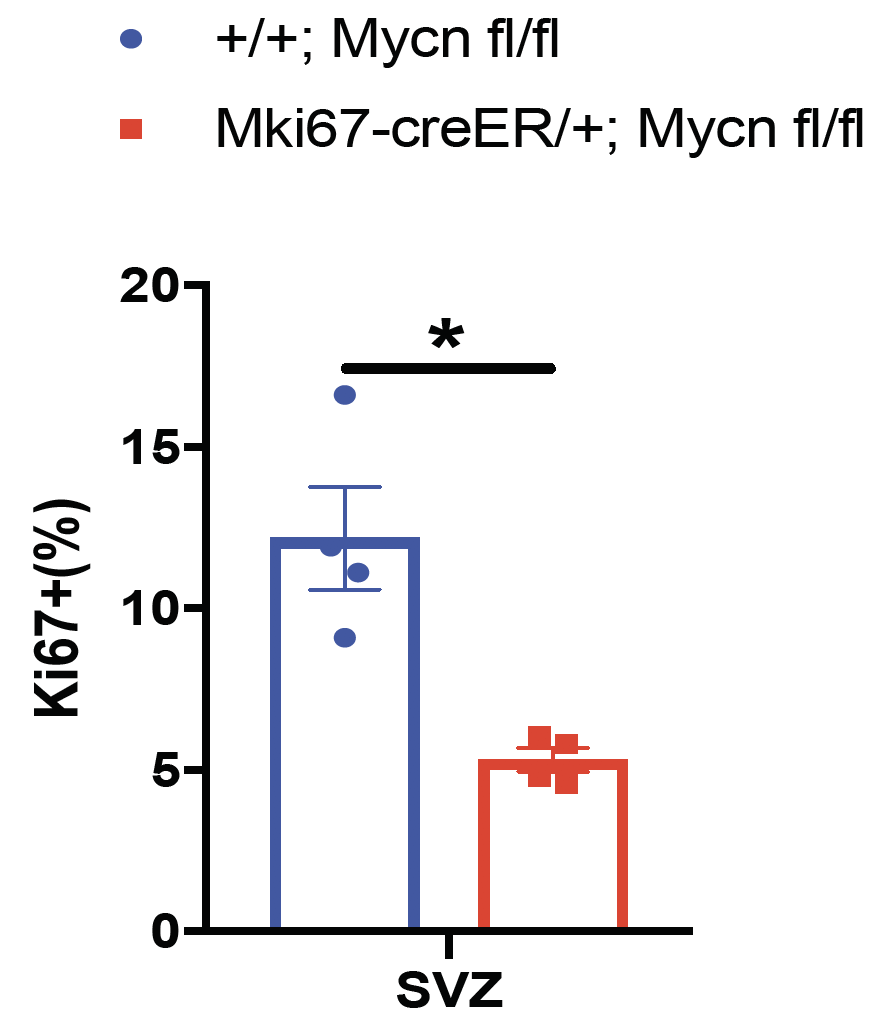


**Supplementary Figure 5. *Mycn* is essential for cell proliferation in SVZ.**

The percentage of Ki67+ cells in the whole cell populations in SVZ decreased significantly in *Mki67-creER/+; Mycn fl/fl* mice compared with that in control *+/+; Mycn fl/fl* mice. Data are shown as Mean ± SEM, and the analysis was unpaired two-tailed Student t-test.


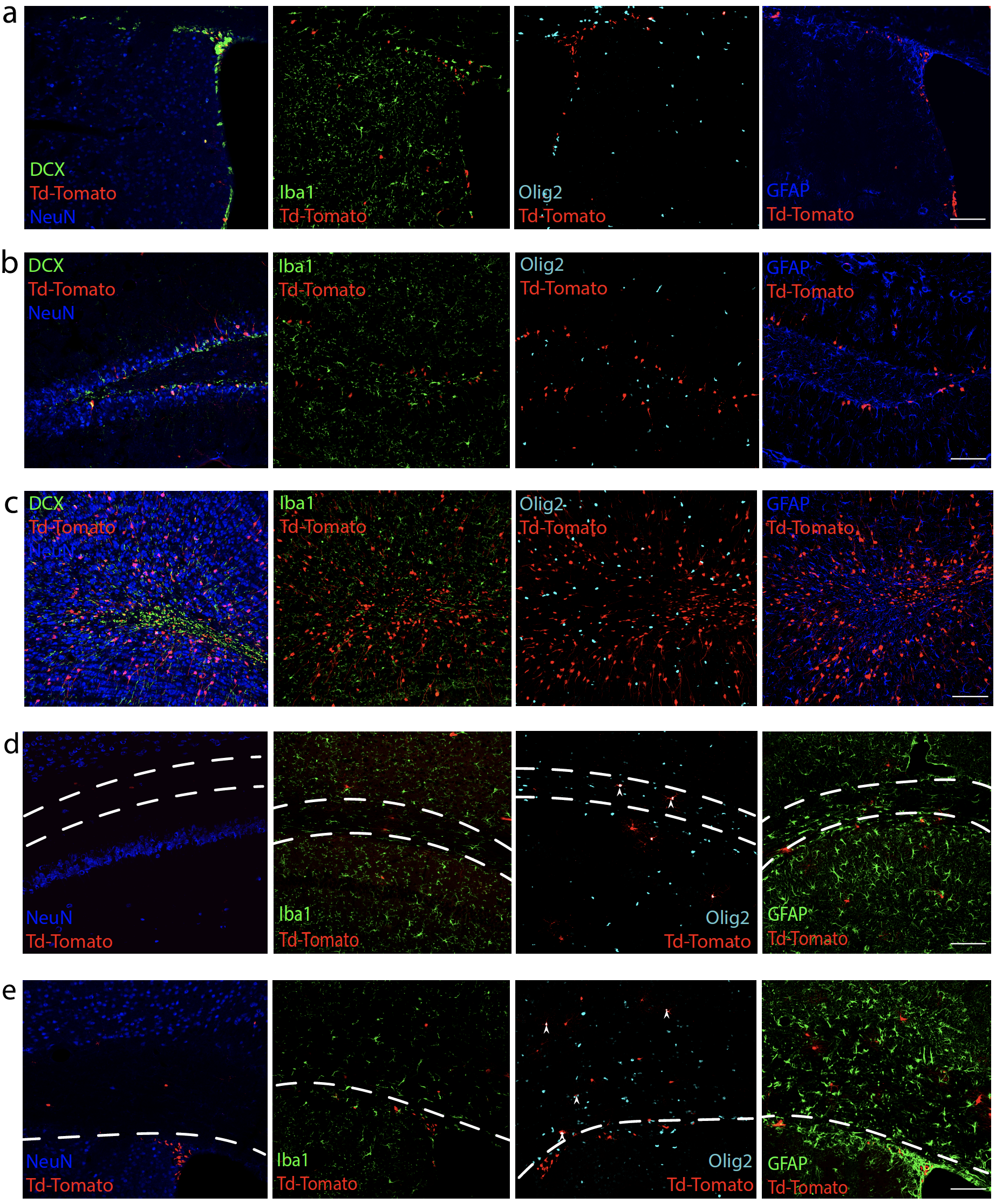


**Supplementary Figure 6. Fate mapping of newborn cells in different brain regions.**

Td-Tomato (+) cells in different brain regions 2 weeks after tamoxifen treatment in young adult *Mki67-creER/+; Td-Tomato/+* mice. In these mice the cells proliferated 2 weeks before were labeled by Td-Tomato. IHC of Td-Tomato and DCX, NeuN, Iba1, Olig2, and GFAP in SVZ (**a**), SGZ (**b**), olfactory bulb (**c**) and SCZ (**d**), and corpus callosum (**e**). The dashed lines in (**d**) show the region of SCZ, and the dashed line in (**e**) marked the boundary between corpus callosum and SVZ. The arrowheads in (**d**) and (**e**) point to Olig2 and Td-Tomato double positive cells in SCZ and corpus callosum. Scale bar = 100 μm.


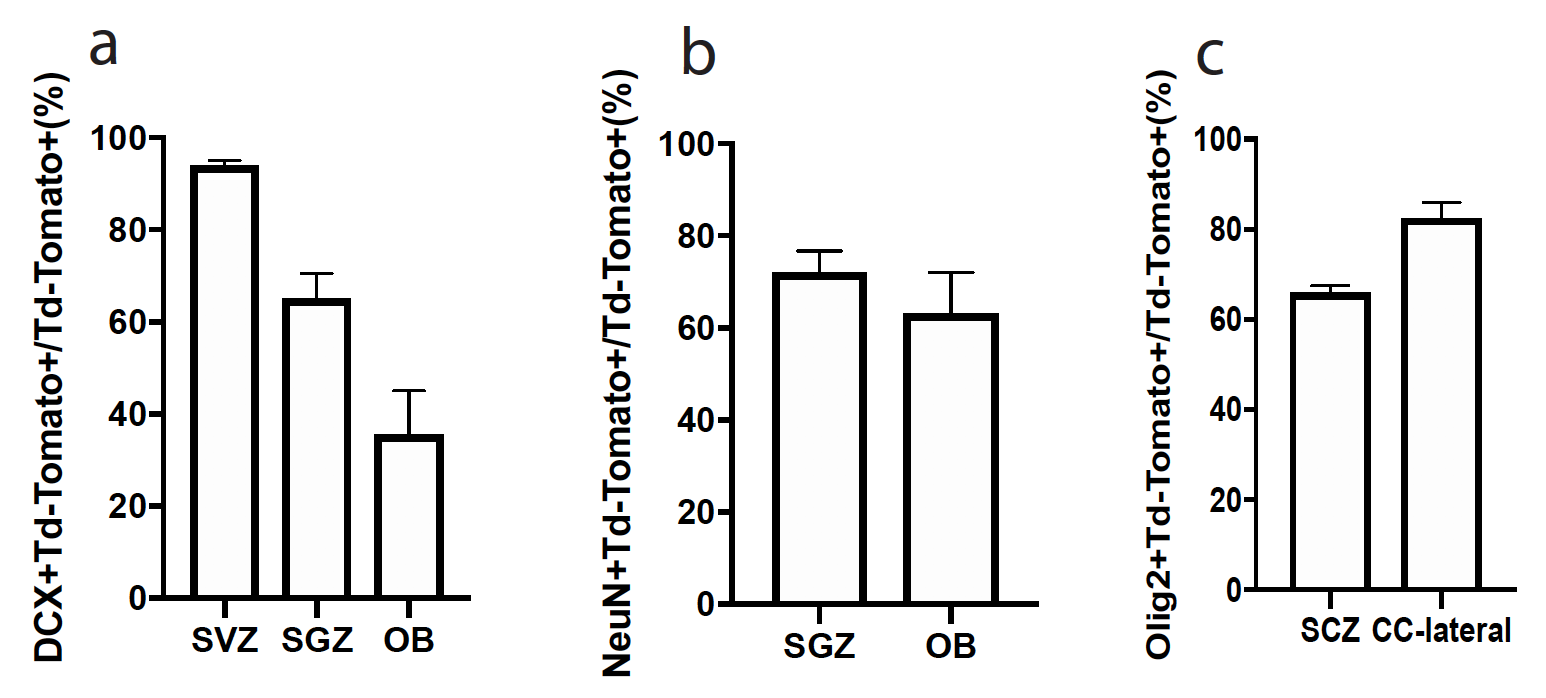


**Supplementary Figure 7. The fate of proliferated cells in different brain regions.**

Quantification of Supplementary Figure 7 on Td-Tomato (+) cells in different brain regions 2 weeks after tamoxifen treatment in young adult *Mki67-creER/+; Td-Tomato/+* mice. In these mice the cells proliferated 2 weeks before were labeled by Td-Tomato.

(**a**) The percentage of DCX/Td-Tomato double positive cells among Td-Tomato (+) cells in SVZ, SGZ and olfactory bulb (OB). (**b**) The percentage of NeuN/Td-Tomato double positive cells among Td-Tomato (+) cells in SGZ and OB.

(**c**) The percentage of Olig2/Td-Tomato double positive cells among Td-Tomato (+) cells in SCZ and corpus callosum (CC).

Data are shown as Mean ± SEM.
